# Prolactin Induces Tuberoinfundibular Dopaminergic Neurone Differentiation in Snell Dwarf Mice if Administered Beginning at 3 Days of Age

**DOI:** 10.1111/j.1365-2826.2009.01869.x

**Published:** 2009-06

**Authors:** C E Khodr, D L Hurley, C J Phelps

**Affiliations:** Neuroscience Program, Tulane University School of MedicineNew Orleans, LA, USA

**Keywords:** tyrosine hydroxylase, feedback regulation, tuberoinfundibular dopaminergic neurones, neurotrophic, prolactin

## Abstract

The hypothalamic tuberoinfundibular dopaminergic (TIDA) neurones secrete dopamine, which inhibits prolactin secretion. TIDA neurone numbers are deficient in Ames (df/df) and Snell (dw/dw) dwarf mice, which lack prolactin, growth hormone and thyroid-stimulating hormone. Prolactin therapy initiated before 21 days maintains normal-sized TIDA neurone numbers in df/df mice and, when initiated as early as 7 days, maintains the maximum TIDA neurone numbers observed in dw/dw development, which are decreased compared to those in normal mice. The present study investigated the effect of prolactin dose and species on TIDA neurone development. Snell dwarf and normal mice were treated with saline, 5 μg of ovine prolactin (oPRL), 50 μg of oPRL, or 50 μg of recombinant mouse prolactin (rmPRL) beginning at 3 days of age. Brains were analysed at 45 days using catecholamine histofluorescence, and immunohistochemistry for tyrosine hydroxylase or bromodeoxyuridine. Normal mice had greater (P ≤ 0.01) TIDA neurones than dw/dw, regardless of treatment. TIDA neurones in 50 μg oPRL-treated dw/dw mice were greater (P ≤ 0.05) than those in 5 μg oPRL- and rmPRL-treated dw/dw mice, which were greater (P ≤ 0.01) than those in saline-treated dw/dw mice. Fifty microgram oPRL-treated dw/dw mice also had greater (P < 0.01) TIDA neurone numbers than the maximum numbers observed in untreated dw/dw mice development. Among saline, 5 μg oPRL and 50 μg oPRL treatments, but not rmPRL, A14 neurone numbers were higher (P ≤ 0.01) in normal compared to in dw/dw mice. The mechanism of TIDA neurone recruitment was investigated using bromodeoxyuridine (BrdU) treatment at intervals after 21 days. Mice treated with rmPRL, but not oPRL, had increased BrdU incorporation in the periventricular area surrounding the third ventricle and median eminence and in the arcuate nucleus. The data obtained in the present study indicate that oPRL, but not rmPRL, when given at a high enough dose, induces TIDA neurone differentiation in dw/dw mice. This supports neurotrophic effects of prolactin on TIDA neurones in early postnatal development that extends beyond maintenance of the cell population.

Prolactin secretion is inhibited by dopamine (1), primarily secreted from tuberoinfundibular dopaminergic (TIDA) neurones. These neurones are located in the arcuate nucleus (ARC) of the hypothalamus and project to the external median eminence (ME) where dopamine is released into the pituitary portal vessels. Prolactin feedback to TIDA neurones increases tyrosine hydroxylase (TH) activity, which is the rate-limiting enzyme in dopamine synthesis (2). Besides dynamic feedback, prolactin also affects the development of TIDA neurones.

The feedback effects of prolactin on TIDA neurones can be studied in models that lack prolactin. Ames (3) and Snell (4) dwarf mice lack pituitary prolactin, growth hormone and thyroid-stimulating hormone due to spontaneous mutation in the gene encoding transcription factor Prop-1 (df/df) or Pit-1 (dw/dw), respectively (5, 6). These mice lack prolactin feedback to TIDA neurones and exhibit a deficient TIDA neurone population (7–9), as well as reduced hypothalamic dopamine (10–12). In Ames dwarf mice, chronic ovine prolactin (oPRL) treatment initiated prior to 21 days of age has been demonstrated to maintain a normal-sized TIDA neurone population (13–15). Snell dwarf mice, however, exhibit an earlier and more severe TIDA neurone deficit (23% of that in normal) than Ames dwarf mice (48% of that in normal). Ames dwarf mice establish a normal-sized TIDA neurone population during development and exhibit a deficient TIDA neurone number by 30 days of age (16). However, Snell dwarf mice never establish a TIDA neurone population comparable to that in normal mice (Dw/dw) and exhibit a deficient TIDA neurone number by 14 days of age (9). Daily oPRL treatment of Snell dwarf mice beginning at 12 or 7 days of age maintains the maximum TIDA neurone numbers observed throughout Snell dwarf development, but does not induce the differentiation of a normal-sized TIDA neurone population (9). Although long-term, homologous prolactin treatment of adult Snell dwarf mice induces differentiation of TIDA neurones (17), the documented effects of prolactin in early postnatal development of Ames and Snell dwarf mice suggest that prolactin inhibits TIDA cell death or loss of phenotype, rather than induces differentiation.

The present study investigated whether the TIDA neurone deficit in Snell dwarf mice could be prevented with early postnatal prolactin therapy. Because of the early and severe TIDA neurone deficit observed in Snell dwarf mice, along with the effect of prolactin on TIDA neurones if therapy is initiated at 7 days of age, prolactin treatment was initiated at 3 days of age in the present study. The effect of dose and species of prolactin on TIDA neurones in Snell dwarf mice also was examined. Snell dwarf and normal mice were treated daily with recombinant mouse prolactin (rmPRL) or oPRL beginning at 3 days of age. Both species of prolactin were hypothesised to induce TIDA neurone differentiation, although rmPRL was hypothesised to be more potent in this effect than oPRL. The mechanism of the hypothesised prolactin-induced recruitment of new TIDA neurones also was investigated using bromodeoxyuridine (BrdU) treatment aiming to assess whether neurogenesis is responsible for increased TIDA neurone number.

## Materials and methods

### Animals

Snell dwarf (dw/dw) and normal (DW/dw) mice were produced from matings of DW/dw females with dw/dw males. Dwarf males were treated with d/l-thyroxine (2 μg, i.p.; Sigma, St Louis, MO, USA) three times a week beginning at 5 weeks of age, and underwent renal capsule pituitary graft surgery from DW/dw donors at 9 weeks of age to induce fertility. Mice undergoing surgery were anaesthetised with isoflurane. Donors, which included DW/dw males and females aged 2–12 months, were anaesthetised with sodium pentobarbital and decapitated for pituitary gland removal. Whole single glands were placed under the kidney capsule of recipient mice. The breeding colony was maintained under a 12 : 12 h light/dark cycle (lights on 06.00 h) and controlled temperature (22 ± 2 °C) with food and water available *ad lib*. Animal use procedures were approved by the Tulane University School of Medicine Animal Care and Use Committee.

### Treatments

DW/dw and dw/dw littermates were treated with saline, 5 μg of oPRL, 50 μg of oPRL or 50 μg of rmPRL (National Hormone and Pituitary Program, National Institute of Diabetes and Digestive and Kidney Diseases), daily, beginning at 3 days of age and continuing until 44 days of age. Litters treated with saline (0.03 m NaHCO_3_/0.15 m NaCl) beginning at 3 days of age served as controls. Treatment solutions were administered subcutaneously for the first 5 days due to solution leakage out of the intraperitoneal space at this age. After the first five treatment days, treatment solution was administered into the intraperitoneal space. Each treatment group contained mice from two or three separate litters. Numbers of mice for each experimental group were: saline (DW/dw, n = 10; dw/dw, n = 6), 5 μg of oPRL (DW/dw, n = 10; dw/dw, n = 11), 50 μg of oPRL (DW/dw, n = 15; dw/dw, n = 14) or 50 μg of rmPRL (DW/dw, n = 3; dw/dw, n = 3). All mice also were treated with BrdU (50 μg/g, i.p.; Sigma) at 21, 31, 39 and 42 days of age and euthanised by perfusion at 45 days of age.

### Tissue preparation and induction of catecholamine fluorescence

At 45 days of age, mice were weighed and anaesthetised with sodium pentobarbital (120 mg/kg body weight). They were then perfused transcardially with 0.9% NaCl followed by 4% paraformaldehyde–0.5% glutaraldehyde (Faglu) fixative (18). After perfusion, brains were removed and postfixed overnight at 4 °C, then transferred to 30% sucrose–Faglu solution to prevent ice crystal formation. Brains were sectioned coronally at 30 μm using a sliding microtome (Reichert; now Leica Microsystems, Wetzlar, Germany), once saturated with sucrose–Faglu. Brain sections were divided into six serial sets, each representative of the entire brain, and post-fixed overnight. Every sixth section (180 μm apart), mounted out of Faglu, was examined for fixative-induced catecholamine fluorescence using narrow-band excitation wavelengths (395–415 nm) and a violet barrier filter (460 nm) on a Nikon E800 microscope (Nikon, Tokyo, Japan) equipped for fluorescence epi-illumination. Remaining sections were stored in cryoprotectant antifreeze (19) at −20 °C.

### Immunohistochemistry for TH or BrdU

Tissue sections taken from cryoprotectant were rinsed in 0.1 m phosphate-buffered saline (PBS), pre-treated with 0.1% hydrogen peroxide to inhibit endogenous peroxidase activity and incubated in 1% aqueous sodium borohydride to reduce glutaraldehyde-fixed linkages and allow antibody access. Sections immunostained for BrdU, but not TH, were incubated in 6 n HCl for 15 min to denature double-stranded DNA. After further rinsing with PBS, sections immunostained for either BrdU or TH were incubated in 1.5% normal rabbit serum for 1 h to reduce nonspecific staining. Sections were then incubated with sheep α-TH (0.025 μg/ml; Pel-Freeze, Rogers, AR, USA) or sheep α-BrdU (0.05 μg/ml; Abcam, Cambridge, MA, USA) for 48 h at room temperature and processed further using biotinylated rabbit α-sheep immunoglobulin G (1 : 200; Vector Laboratories, Burlingame, CA, USA) and avidin–biotin complex solutions (Vectastain kit; Vector Laboratories). Development with 0.02% diaminobenzedine tetrahydrochloride (DAB) and 0.003% H_2_O_2_ in Tris buffer allowed visualisation of immunoreactivity. 0.5% NiSO_4_ was added to the DAB solution for visualisation of BrdU immunoreactivity. Sections were then mounted, dried and coverslipped using DPX mountant for microscopy (Fisher Chemical Co., Pittsburgh, PA, USA). Each immunohistochemistry run used identical antiserum aliquots and reagents.

### TH cell counts

TH-immunoreactive neurones in hypothalamic dopaminergic areas A12 (TIDA), A13 (zona incerta) and A14 (periventricular nucleus), according to the classification of Björklund and Nobin (20), were manually quantified at 180-μm intervals. Cell counts were not corrected for re-counted or missed cells because section thickness (30 μm) and interval (180 μm) exceeded perikaryal diameter (21), but cell counts were corrected for periodicity (× 6, for every sixth section).

### BrdU cell counts

BrdU-immunoreactive cells were manually quantified at × 40 magnification in the subventricular zone (SVZ) of the lateral ventricle (LV), the dentate gyrus (DG), the periventricular region surrounding the third ventricle (3V) and ME, and the ARC. BrdU-immunoreactive cells in the entire SVZ were quantified beginning at the wall of the LV and extending approximately 25 μm into the surrounding tissue. BrdU-immunoreactive cells in the periventricular region surrounding the 3V were quantified beginning at the wall of the 3V and extending approximately 60 μm into the surrounding tissue, excluding the ARC. Sample sizes for BrdU cell counts were: saline (DW/dw, n = 5; dw/dw, n = 5), 5 μg of oPRL (DW/dw, n = 5; dw/dw, n = 5), 50 μg of oPRL (DW/dw, n = 5; dw/dw, n = 6) or 50 μg of rmPRL (DW/dw, n = 3; dw/dw, n = 5). Cell counts were corrected for periodicity (× 6, for every sixth section).

### Imaging for illustrations

A DX1200 colour digital camera on a Nikon E800 microscope was used to capture images of endogenous catecholamine fluorescence, TH immunostaining and BrdU immunostaining. Images were composited into multi-photo plates using Adobe Photoshop 7.0 (Adobe Systems, San Jose, CA, USA).

### Statistical analysis

anova (Superanova; Abacus Concepts, Berkeley, CA, USA) and Student Newman–Keuls post-hoc tests were used to statistically analyse data. Two-way anova was initially used to identify significant effects. If significant effects were observed in the two-way anova, one-way anova was performed with post-hoc tests. P < 0.05 was considered statistically significant.

## Results

### Body weight

**Fig. 1:**
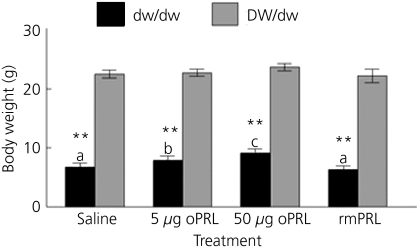
Body weights of Snell dwarf (dw/dw; black columns) and normal (DW/dw; grey columns) mice treated with saline, 5 μg of ovine prolactin (oPRL), 50 μg of oPRL or 50 μg of recombinant mouse prolactin (rmPRL) beginning at 3 days of age. Data are the mean ± SEM. Sample size for each experimental group: saline (DW/dw, n = 10; dw/dw, n = 6), 5 μg of oPRL (DW/dw, n = 10; dw/dw, n = 11), 50 μg of oPRL (DW/dw, n = 15; dw/dw, n = 14) and 50 μg of rmPRL (DW/dw, n = 3; dw/dw, n = 3). Asterisks indicate differences between dwarf and normal (**P < 0.01). Letters indicate differences between treatments (a and b, b and c differ by P < 0.05; a and c differ by P < 0.01).

Body weights of DW/dw and dw/dw treated daily with saline, 5 μg of oPRL, 50 μg of oPRL or 50 μg of rmPRL beginning at 3 days of age are shown in Fig. 1. There was an interaction of sex and genotype on body weight (F_1,68_ = 4.5, P = 0.0369). Sex affected body weight in DW/dw (F_1,36_ = 11.5, P = 0.0017), but not in dw/dw; male DW/dw (23.8 ± 0.4 g, n = 24) weighed more (P ≤ 0.01) than female DW/dw (21.5 ± 0.6 g, n = 14). Although sex affected body weight in DW/dw, genders were pooled because there was a parallel insignificant effect of treatment on male and female DW/dw. Treatment significantly affected body weight in dw/dw (F_3,30_ = 18.0, P = 0.0001), but not in DW/dw. Dwarf mice treated with 50 μg of oPRL (9.1 ± 0.3 g, n = 14) weighed more (P ≤ 0.05) than dw/dw treated with 5 μg of oPRL (7.9 ± 0.2 g, n = 11), which weighed more (P ≤ 0.05) than dw/dw treated with saline (6.7 ± 0.2 g, n = 6). Linear regression analysis showed a dose-dependent effect of oPRL treatment (r = 0.9999) on body weight in dw/dw. Dwarfs treated with 5 μg of oPRL weighed more (P ≤ 0.01) than dw/dw treated with rmPRL (6.3 ± 0.3 g, n = 3), which had similar weights to dw/dw treated with saline. Genotype had an effect on body weight, regardless of treatment (F_1,70_ = 1122.8, P = 0.0001). In all treatment groups, dw/dw (8.1 ± 0.2 g, n = 34) weighed less (P ≤ 0.01) than DW/dw (22.9 ± 0.4 g, n = 38).

### Catecholamine fluorescence

**Fig. 2:**
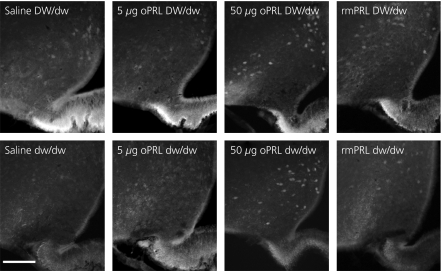
Endogenous catecholamine fluorescence in tuberoinfundibular dopaminergic cell bodies and median eminence of Snell dwarf (dw/dw, lower panels) and normal (DW/dw, upper panels) mice treated daily with saline, 5 μg of ovine prolactin (oPRL), 50 μg of oPRL or 50 μg of recombinant mouse prolactin (rmPRL) beginning at 3 days of age. Coronal sections; original objective magnification: × 20. Scale bar = 100 μm.

Figure 2 illustrates catecholamine fluorescence in TIDA neurones and ME of normal (DW/dw) and Snell dwarf (dw/dw) mice treated daily with saline, 5 μg of oPRL, 50 μg of oPRL, or 50 μg of rmPRL beginning at 3 days of age. Catecholamine fluorescence was qualitatively evaluated. Among DW/dw, TIDA neurone fluorescence in 50 μg oPRL-treated mice was elevated compared to that in rmPRL-treated mice, which was elevated compared to that in 5 μg oPRL- or saline-treated mice. ME fluorescence was similar in all DW/dw treatment groups. Dwarf mice treated with 5 or 50 μg of oPRL had slightly higher ME fluorescence than dw/dw treated with saline or rmPRL. rmPRL-treated dwarf mice had slightly higher TIDA fluorescence than saline and 5 μg oPRL-treated dw/dw and lower TIDA fluorescence than 50 μg oPRL-treated dw/dw. In saline-, 5 μg oPRL-, and rmPRL-treated mice, DW/dw TIDA perikaryal and ME fluorescence was increased compared to that in dw/dw. Normal and dwarf mice treated with 50 μg of oPRL had similar TIDA neurone fluorescence, but ME fluorescence in DW/dw was elevated compared to that in dw/dw. Dopamine fluorescence in nonhypophysiotrophic regions was similar between genotypes and treatments (data not shown).

### TH immunoreactivity

**Fig. 3:**
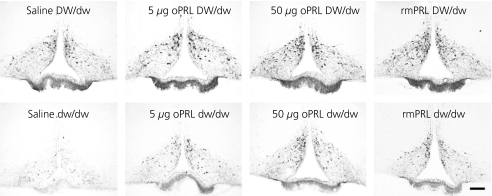
TH immunoreactivity in tuberoinfundibular dopaminergic neurones and median eminence of Snell dwarf (dw/dw, lower panels) and normal (DW/dw, upper panels) mice treated daily with saline, 5 μg of ovine prolactin (oPRL), 50 μg of oPRL or 50 μg of recombinant mouse prolactin (rmPRL) beginning at 3 days of age. Coronal sections; original objective magnification: × 10. Scale bar = 100 μm.

ARC and ME TH immunoreactivity in DW/dw and dw/dw treated daily with saline, 5 μg of oPRL, 50 μg of oPRL or 50 μg of rmPRL, beginning at 3 days of age, are shown in Fig. 3. TH immunoreactivity was qualitatively similar in DW/dw regardless of treatment. A qualitative dose dependent effect of oPRL was seen in dw/dw, with saline-treated dw/dw having the least TH immunoreactivity and 50 μg oPRL-treated dw/dw having the most TH immunoreactivity. TH immunostaining in dw/dw treated with rmPRL was similar to that in dw/dw treated with 5 μg of oPRL. All dw/dw mice had reduced TH immunostaining in TIDA neurones and ME compared to DW/dw. Immunostaining for TH in nonhypophysiotrophic regions was qualitatively similar between genotypes and treatments (data not shown).

**Fig. 4:**
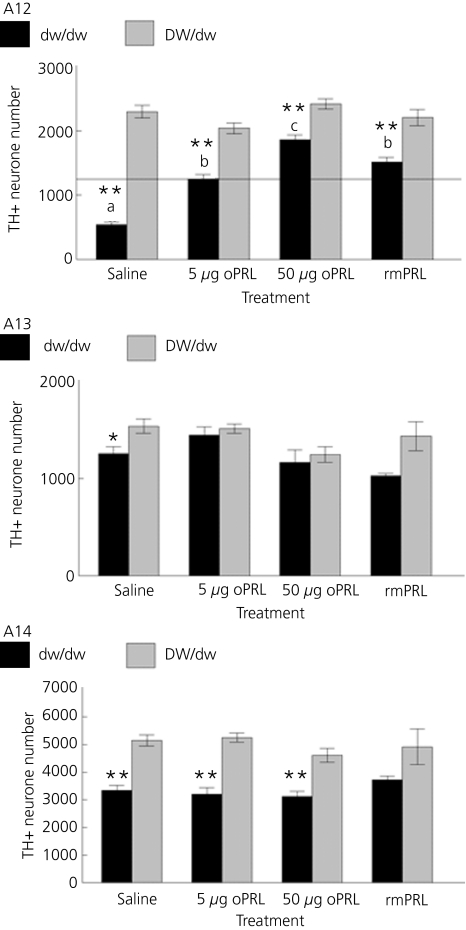
Tyrosine hydroxylase-immunoreactive neurone numbers in areas A12 (top), A13 (middle), and A14 (bottom) of Snell dwarf (dw/dw, black bars) and normal (DW/dw, grey bars) mice treated daily with saline, 5 μg of ovine prolactin (oPRL), 50 μg of oPRL or 50 μg of recombinant mouse prolactin (rmPRL) beginning at 3 days of age. Data are the mean ± SEM. Asterisks represent differences between dwarf and normal (*P < 0.05, **P < 0.01). Letters represent differences between treatments (a and b, a and c differ by P < 0.01; b and c differ by P < 0.05). The horizontal line in the top graph represents the maximum number of tuberoinfundibular dopaminergic neurones observed throughout untreated Snell dwarf development (7–21 days postnatally) as reported previously (9).

Neurone numbers immunoreactive for TH in areas A12, A13 and A14 of DW/dw and dw/dw treated daily with saline, 5 μg of oPRL, 50 μg of oPRL or 50 μg of rmPRL, beginning at 3 days of age, are shown in Fig. 4. Genders were pooled in saline, 5 μg of oPRL, and 50 μg of oPRL treatment groups because three-way anova indicated no effect of sex on neurone number in any area examined for TH immunoreactivity. There was a significant effect of treatment on area A12 neurone number in dw/dw (F_4,30_ = 74.1, P = 0.0001), but not in DW/dw. Dwarfs treated with 50 μg of oPRL (1857 ± 55, n = 14) had higher TIDA neurone numbers (P ≤ 0.01) than dw/dw treated with 5 μg of oPRL (1252 ± 40, n = 11), which had higher (P ≤ 0.01) TIDA neurone numbers than dw/dw treated with saline (538 ± 37, n = 6). Linear regression analysis showed that oPRL had a dose-dependent effect (r = 0.9989) on area A12 neurone number in dw/dw. TIDA neurone numbers in dw/dw treated with rmPRL (1514 ± 63, n = 3) were similar to those in dw/dw treated with 5 μg of oPRL, but lower (P ≤ 0.05) than those in dw/dw treated with 50 μg of oPRL. Furthermore, TIDA neurone number in 50 μg of oPRL-treated dw/dw was greater (P ≤ 0.01) than the highest TIDA neurone numbers observed in untreated dw/dw development (7–21 days; 1295 ± 139, n = 6; horizontal line in Fig. 4). Dwarfs treated with 5 μg of oPRL or rmPRL had TIDA neurone numbers that were similar to the highest numbers observed throughout dw/dw development (7–21 days of age, horizontal line in Fig. 4). Genotype significantly affected TIDA neurone number (F_1,71_ = 80.4, P = 0.0001), regardless of treatment. In all treatments, DW/dw (2270 ± 53, n = 38) had higher (P ≤ 0.01) A12 neurone numbers than dw/dw (1398 ± 87, n = 34). A one-way anova combining treatment and phenotype indicated that A12 neurone numbers in 50 μg of oPRL-treated dw/dw were similar to those in saline (2296 ± 96, n = 10), 5 μg oPRL (2039 ± 89, n = 10) and rmPRL-treated DW/dw (2204 ± 124, n = 3).

Area A13 neurone number was not affected by treatment. Saline-treated A13 neurone number was affected by genotype (F_1,14_ = 6.6, P = 0.0221); saline-treated DW/dw had higher (P ≤ 0.05) A13 neurone numbers (1539 ± 74, n = 10) than saline-treated dw/dw (1256 ± 72, n = 6). However, a one-way anova that combined genotype and treatment indicated no differences in A13 neurone numbers between genotypes and treatments. Because the one-way anova combining treatment and genotype indicated no differences in A13 neurone number, A13 neurone number was considered comparable between groups. Area A14 neurone number was not affected by treatment but was significantly affected by genotype (F_1,71_ = 96.1, P = 0.0001). A14 neurone numbers were higher (P ≤ 0.01) in DW/dw (4946 ± 138, n = 35) than in dw/dw (3198 ± 120, n = 31) in saline, 5 μg of oPRL and 50 μg of oPRL treatments, but not in the rmPRL treatment. One-way anova combining genotype and treatment (F_8,64_ = 13.0, P = 0.0001) indicated that A14 neurone numbers in rmPRL-treated dw/dw (3720 ± 134, n = 3) were similar to those in 50 μg oPRL-treated DW/dw (4611 ± 249, n = 15), but lower (P ≤ 0.05) than those in saline- (5147 ± 201, n = 10) and 5 μg oPRL-treated DW/dw (5249 ± 162, n = 10).

### BrdU immunoreactivity

**Fig. 5:**
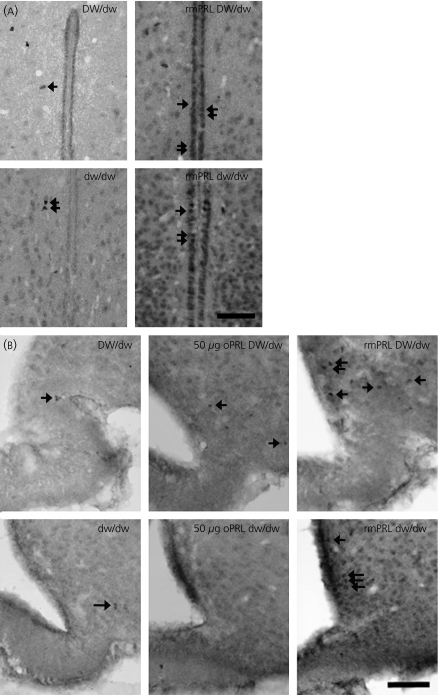
(a) Bromodeoxyuridine (BrdU) immunoreactivity in the periventricular region surrounding the third ventricle of Snell dwarf (dw/dw, lower panels) and normal (DW/dw, upper panels) mice treated with saline, 5 μg of ovine prolactin (oPRL), 50 μg of oPRL (grouped in left panels) or 50 μg of recombinant mouse prolactin (rmPRL) (right panels) beginning at 3 days of age. Coronal sections; original objective magnification: × 40. Scale bar = 50 μm. (b) BrdU immunoreactivity in the median eminence and arcuate nucleus of Snell dwarf (dw/dw, lower panels) and normal (DW/dw, upper panels) mice treated with saline, 5 μg of oPRL (grouped in left panels), 50 μg of oPRL (middle panels) or 50 μg of rmPRL (right panels) beginning at 3 days of age. Coronal sections; original objective magnification: × 40 . Scale bar = 50 μm.

Figure 5(a) shows representative illustrations of BrdU immunoreactivity in the periventricular region surrounding the 3V of DW/dw and dw/dw treated daily with saline, 5 μg of oPRL, 50 μg of oPRL (grouped in figure) or 50 μg of rmPRL beginning at 3 days of age. Within genotype, immunoreactivity for BrdU was similar between saline, 5 μg of oPRL and 50 μg of oPRL treatments (not shown) in the periventricular region surrounding the 3V. However, 3V BrdU immunostaining in rmPRL-treated mice was qualitatively greater than that in all other treatments. BrdU incorporation was greater in DW/dw than in dw/dw in all treatments.

BrdU immunoreactivity in the ARC of DW/dw and dw/dw treated daily with saline, 5 μg of oPRL (grouped in figure), 50 μg of oPRL or 50 μg of rmPRL beginning at 3 days of age, is shown in Fig. 5(b). Within genotype, mice treated with rmPRL exhibited greater BrdU incorporation than those treated with saline, 5 μg of oPRL, or 50 μg of oPRL, which had qualitatively similar BrdU immunoreactivity. In mice treated with 50 μg of oPRL or rmPRL, DW/dw had greater BrdU immunoreactivity than dw/dw. Dentate gyrus and SVZ BrdU immunoreactivity was qualitatively similar between treatments and genotypes (not shown).

**Fig. 6:**
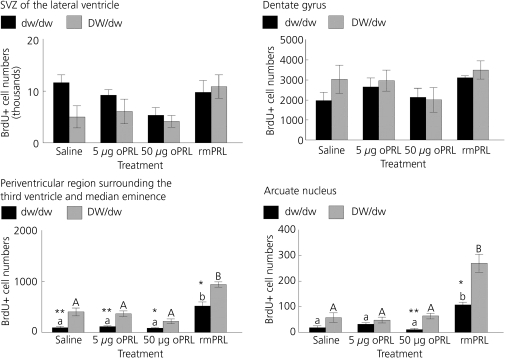
Bromodeoxyuridine (BrdU)-immunoreactive cell numbers in the SVZ of the lateral ventricle, the dentate gyrus, the periventricular region surrounding the third ventricle and the median eminence, and the arcuate nucleus of Snell dwarf (dw/dw, black bars) and normal (DW/dw, grey bars) mice treated with saline, 5 μg of ovine prolactin (oPRL), 50 μg of oPRL or 50 μg of recombinant mouse prolactin (rmPRL) beginning at 3 days of age. Data are the mean ± SEM. Asterisks indicate differences between dwarf and normal (*P < 0.05, **P < 0.01). Letters indicate differences between treatments. Capitalised letters represent differences between normal mice; lower case letters represent differences between mice. Bars labelled with ‘A’ or ‘a’ differ from bars labelled with ‘B’ or ‘b’ by P < 0.01.

Figure 6 shows BrdU-immunoreactive cell numbers in the SVZ of the LV, the DG, the periventricular region surrounding the 3V and ME, and the ARC of DW/dw and dw/dw treated daily with saline, 5 μg of oPRL, 50 μg of oPRL or 50 μg of rmPRL beginning at 3 days of age. Three-way anova indicated no effect of sex on any area examined for BrdU immunoreactivity in mice treated with saline, 5 μg of oPRL, or 50 μg of oPRL, so genders were pooled.

Cell numbers immunoreactive for BrdU in the DG and SVZ of the LV were not affected by treatments or genotypes. In the periventricular region surrounding the 3V and ME, an effect of treatment was observed (F_3,33_ = 15.1, P = 0.0001) in DW/dw and dw/dw. Mice treated with rmPRL had higher (P ≤ 0.01) 3V periventricular and ME BrdU-immunoreactive cell numbers (728 ± 104, n = 6) than mice treated with saline (250 ± 65, n = 10), 5 μg of oPRL (239 ± 52, n = 10) or 50 μg of oPRL (147 ± 30, n = 11). Genotype also affected BrdU incorporation in the periventricular region surrounding the 3V and ME (F_1,35_ = 12.9, P = 0.0010) regardless of treatment; BrdU-immunoreactive cell numbers were higher (P ≤ 0.01) in DW/dw (431 ± 65, n = 18) than in dw/dw (164 ± 39, n = 19) in all treatments. A one-way anova combining genotype and treatment (F_7,29_ = 30, P = 0.0001) indicated that rmPRL-treated dw/dw had similar 3V and ME BrdU-immunoreactive cell numbers (516 ± 81, n = 3) to DW/dw treated with saline (404 ± 80, n = 5) and 5 μg of oPRL (366 ± 59, n = 5), and higher (P ≤ 0.01) BrdU-immunoreactive cell numbers than DW/dw treated with 50 μg of oPRL (219 ± 48, n = 5). This one-way anova also indicated that saline (96 ± 23, n = 5), 5 μg oPRL (112 ± 16, n = 5) and 50 μg oPRL-treated dw/dw (87 ± 15, n = 6) had similar BrdU-immunopositive cell numbers to DW/dw treated with 50 μg of oPRL, and lower cell numbers than DW/dw treated with rmPRL (940 ± 50, n = 3), saline or 5 μg of oPRL.

In the ARC, an effect of treatment was observed (F_3,33_ = 17.4, P = 0.0001) on BrdU incorporation regardless of genotype. In both DW/dw and dw/dw, rmPRL-treated mice had higher (P ≤ 0.01) BrdU immunoreactive cell numbers (189 ± 40, n = 6) than saline- (39 ± 11, n = 10), 5 μg oPRL- (41 ± 7, n = 10) or 50 μg oPRL-treated mice (35 ± 10, n = 11). An effect of genotype also was observed (F_1,35_ = 6.911, P = 0.0126) on ARC BrdU immunoreactivity; 50 μg oPRL-treated DW/dw had higher (P ≤ 0.01) BrdU-immunoreactive cell numbers (64 ± 12, n = 5) than 50 μg oPRL-treated dw/dw (11 ± 5, n = 6), and rmPRL-treated DW/dw had higher (P ≤ 0.05) BrdU-immunoreactive cell numbers (270 ± 36, n = 3) than rmPRL-treated dw/dw (108 ± 11, n = 3). A one-way anova combining genotype and treatment (F_7,29_ = 32.1, P = 0.0001) indicated that rmPRL-treated dw/dw had higher (P ≤ 0.05) BrdU-immunoreactive cell numbers than saline- (58 ± 18, n = 5), 5 μg oPRL- (48 ± 11, n = 5), and 50 μg oPRL-treated DW/dw.

## Discussion

PRL feedback regulates the TIDA neurone population by inducing the expression of TH (2) and increasing dopamine synthesis and release (1). However, the effects of prolactin on TIDA neurones extend beyond this dynamic regulation. Studies from this laboratory have shown that prolactin can act as a neurotrophhic factor on the TIDA neurone population (22). Treatment of Ames (13, 14) or Snell (9) dwarf mice with daily prolactin beginning at 12 days of age maintains the maximum TIDA neurone numbers observed throughout untreated dwarf development. This is a normal-sized neurone population in Ames dwarf mice because Ames dwarf mice establish a normal-sized TIDA neurone population by 21 days of age, after which cell numbers decline to 48% of that in normal animals (8). However, this is a deficient neurone population in Snell dwarf mice because Snell dwarf mice never establish a normal-sized TIDA neurone number and exhibit a deficit of 23% of that in normal animals (9). Thus, previous studies investigating the early postnatal effect of prolactin on TIDA neurones in Ames and Snell dwarf mice suggest that prolactin only maintains the survival of existing TIDA neurones (23).

The present study was undertaken to investigate whether the development of a normal-sized TIDA neurone population could be induced in response to prolactin in Snell dwarf mice. Because of the early and severe TIDA neurone deficit observed in Snell dwarf mice (9), prolactin treatment was initiated at 3 days of age. The effect of dose and species of prolactin was investigated. As in previous studies, the present results show that prolactin acts as a neurotrophic factor on TIDA neurones in Snell dwarf mice. Unlike in Ames dwarf mice, however, prolactin was shown to induce differentiation of TIDA neurones, rather than solely promote survival of the maximum numbers seen in development. The high dose (50 μg) of oPRL induced differentiation of a near-normal TIDA neurone population in Snell dwarf mice, which never exhibit TIDA neurone numbers of this quantity during development. The low (5 μg) oPRL dose only maintained the maximum TIDA neurone numbers seen in Snell dwarf development (7–21 days; Fig. 4).

The effect of rmPRL on TIDA neurone differentiation was hypothesised to be more potent than the observed heterologous prolactin effects because murine and ovine prolactin are only 56% homologous (24). It was therefore postulated that oPRL would have a lower total active fraction in mouse tissues than homologous prolactin. However, the present study showed that the effect of 50 μg of rmPRL on TIDA neurone differentiation was as potent as that of the low (5 μg) oPRL dose; therefore, rmPRL did not induce TIDA neurone differentiation. It is possible that rmPRL has an inverse effect on TIDA neurone number; thus, a lower dose of homologous prolactin may have a greater effect on TIDA neurone differentiation. Alternatively, oPRL may have properties that homologous prolactin lacks. The current data indicate a somatogenic effect of oPRL that is not observed with homologous prolactin. Dwarf mice treated with oPRL gained weight in a direct dose-dependent manner, but dwarf mice treated with homologous prolactin had similar weights to those treated with saline (Fig. 1). This oPRL-induced weight gain does not definitively indicate somatogenic characteristics of oPRL because the weight gain may be attributed to adipose gain, a known effect of prolactin, rather than long bone growth. However, oPRL has been shown to induce liver insulin-like growth factor-1 gene expression in hypophysectomised rats (25), which supports somatogenic characteristics of oPRL. Other possibilities for the observed differential effects of oPRL and rmPRL on TIDA neurones may include differences in clearance or differences in transport into the brain. A comparison of plasma prolactin levels could not be assessed because the majority of plasma samples were lost in the aftermath of Hurricane Katrina.

The present data clearly indicate that prolactin acts as a neurotrophic factor on TIDA neurones. Both heterologous and homologous prolactin induced survival of the greatest TIDA neurone numbers observed in untreated Snell dwarf development. This effect suggests that prolactin inhibits TIDA cell death or phenotype change, resulting in a less severe TIDA deficit. Although the high dose of heterologous prolactin was able to induce differentiation of TIDA neurone numbers greater than those ever seen in Snell dwarf development, the TIDA deficit was not prevented.

Differentiation of a TIDA neurone population that is larger than that ever seen in untreated dwarf development in response to a high enough dose of heterologous prolactin was demonstrated in the present study. The observed new TIDA neurones could possibly be recruited through two mechanisms, including neurogenesis and/or differentiation of pre-existing neurones. Although hypothalamic neurogenesis is only thought to occur prenatally, there is *in vitro* evidence indicating the postnatal existence of quiescent hypothalamic progenitor cells. These cells, as well as hippocampal progenitors, have been shown to differentiate into multiple neuroendocrine phenotypes *in vitro* (26). These data indicate that hypothalamic neurogenesis could possibly occur postnatally if the quiescent progenitor cells are appropriately stimulated, or if hippocampal progenitor cells differentiate and migrate to the hypothalamus. Indeed, BrdU-immunoreactive neurones have been detected in the hypothalamus in response to brain-derived neurotrophic factor (27) and ciliary neurotrophic factor (28).

In the present study, the neurogenic effects of prolactin were investigated as a mechanism for the increased TIDA neurone numbers. Shingo *et al.* (29) have previously demonstrated neurogenic effects of prolactin in the SVZ of the LV in both male and female adult mice, resulting in new olfactory interneurones. The results obtained in the present study indicated treatment and genotype effects on BrdU incorporation in the periventricular region surrounding the 3V and median eminence, and in the ARC, but not in the SVZ of the LV or in the DG.

Although the present study indicated no effect of treatment or genotype on BrdU incorporation in the SVZ of the LV, there was an insignificant trend of decreased SVZ BrdU incorporation in response to increasing oPRL levels in dwarf mice. An inverse insignificant trend of oPRL dose and SVZ BrdU immunoreactive cell numbers observed in the present study conflicts with the study by Shingo *et al.* (29). However, Shingo *et al.* (29) demonstrated that rmPRL, not oPRL, induced SVZ neurogenesis in normal mice. Although not significant, treatment of normal mice with rmPRL, but not oPRL, induced an increased trend of BrdU incorporation in the SVZ of the LV, which is in accordance with the results obtained by Shingo *et al.* (29). The lack of an observed effect of prolactin treatment in normal mice may be due to differences in treatment in the present study compared with that of Shingo *et al.* (29). Shingo *et al.* (29) demonstrated that treatment of normal mice with rmPRL for 6 days induces SVZ BrdU incorporation and that these cells further migrate to the olfactory bulb. The treatments in the present study lasted for 42 days and BrdU incorporation was not quantified in the olfactory bulb. It is possible that the initial short-term effect of prolactin is induction of SVZ cell proliferation, after which the new cells migrate to the olfactory lobe.

Genotype differences were consistent between treatments in the periventricular region surrounding the 3V and ME; dwarfs experienced less BrdU incorporation compared to normal mice. In the ARC, there were no genotype differences in saline and 5 μg of oPRL-treated mice, but dwarfs treated with 50 μg of oPRL or rmPRL had lower BrdU incorporation. In both dwarfs and normals, rmPRL, but not oPRL, induced increased BrdU incorporation in the periventricular region surrounding the 3V and ME and in the ARC (Fig. 6).

These results suggest that rmPRL, but not oPRL, induces cell proliferation in the periventricular region surrounding the 3V and ME, and the ARC in both dwarf and normal mice. It is unlikely that the increased ARC cell proliferation contributes to the observed increase in TIDA neurone number because mice treated with rmPRL did not exhibit higher TIDA neurone numbers than those seen in untreated dwarf development (Fig. 4). However, mice treated with 50 μg of oPRL exhibited greater TIDA neurone numbers than those seen in untreated dwarf development, but did not experience increased BrdU incorporation in any area. Because BrdU treatment was not initiated until 21 days of age, the mechanism of TIDA neurone recruitment through neurogenesis cannot be excluded. It is possible that new neurones are recruited for differentiation to the TIDA phenotype prior to 21 days of age. Alternatively, the increased TIDA neurone population could result from an induced phenotype change in neighbouring ARC neurones. Because the ARC contains a heterogeneous neurone population, a wide variety of neurone types might comprise the target of this phenotype change.

There is evidence that dopamine secreted by the A14 neurone population participates in regulation of prolactin secretion (30). Previous studies also have indicated an effect of prolactin on A14 neurone activity (31). In the present study, a deficient A14 neurone number was observed in Snell dwarf mice, confirming previous reports from this laboratory (17). This observed A14 neurone deficit suggests a possible neurotrophic role of prolactin on this neurone population. The present study indicated that the A14 neurone deficit was not affected by heterologous prolactin. Although homologous prolactin treatment of Snell dwarf mice was shown to statistically induce near-normal A14 neurone numbers (comparable to those in normal mice treated with 50 μg of oPRL or rmPRL), A14 neurone number was not different between dwarfs. Because of the small number of mice treated with homologous prolactin (DW/dw, n = 3; dw/dw, n = 3), as well as the A14 cell number variation in the rmPRL-treated normal mice, the observed effects of homologous prolactin on A14 cell numbers in dwarfs may not be conclusive. However, in our previous studies, we did not observe induction of A14 neurone differentiation in response to long-term, homologous prolactin (17). The present data further support a neurotrophic role for prolactin on A14 neurones and suggest involvement of prolactin and/or other factors (such as growth hormone and/or thyroid-stimulating hormone), possibly in conjunction with prolactin, in A14 neurone development.

In summary, the present study has demonstrated the promotion of a near normal-sized TIDA neurone population in response to a sufficiently high dose of heterologous prolactin, suggesting that prolactin induces differentiation and/or proliferation of TIDA neurones. Because treatment did not affect BrdU-immunoreactive cell numbers, the demonstrated prolactin-induced TIDA neurone recruitment cannot be attributed to prolactin-induced neurogenesis. Neurogenesis as a mechanism responsible for the increased TIDA neurone numbers, however, cannot be excluded because BrdU treatment was not administered for the full course of prolactin therapy. A dose-dependent effect of heterologous prolactin on TIDA neurone numbers also was demonstrated. Both homologous prolactin and the low dose of heterologous prolactin only maintained the greatest TIDA neurone numbers observed in Snell dwarf development, but did not induce TIDA neurone differentiation. The results obtained in the present study also confirm previous results that demonstrate a possible neurotrophic effect of prolactin on A14 neurones.
